# The neuroprotective effect of dexmedetomidine and its mechanism

**DOI:** 10.3389/fphar.2022.965661

**Published:** 2022-09-20

**Authors:** Yijun Hu, Hong Zhou, Huanxin Zhang, Yunlong Sui, Zhen Zhang, Yuntao Zou, Kunquan Li, Yunyi Zhao, Jiangbo Xie, Lunzhong Zhang

**Affiliations:** ^1^ Neurology Department, Weifang Hospital of Traditional Chinese Medicine, Weifang, China; ^2^ Graduate School, Shandong University of Traditional Chinese Medicine, Jinan, China

**Keywords:** dexmedetomidine, neuroprotective, inflammatory response, cell apoptosis, blood-brain barrier, cell structure protection, autophagy

## Abstract

Dexmedetomidine (DEX) is a highly selective α2 receptor agonist that is routinely used in the clinic for sedation and anesthesia. Recently, an increasing number of studies have shown that DEX has a protective effect against brain injury caused by traumatic brain injury (TBI), subarachnoid hemorrhage (SAH), cerebral ischemia and ischemia–reperfusion (I/R), suggesting its potential as a neuroprotective agent. Here, we summarized the neuroprotective effects of DEX in several models of neurological damage and examined its mechanism based on the current literature. Ultimately, we found that the neuroprotective effect of DEX mainly involved inhibition of inflammatory reactions, reduction of apoptosis and autophagy, and protection of the blood–brain barrier and enhancement of stable cell structures in five way. Therefore, DEX can provide a crucial advantage in neurological recovery for patients with brain injury. The purpose of this study was to further clarify the neuroprotective mechanisms of DEX therefore suggesting its potential in the clinical management of the neurological injuries.

## Introduction

Dexmedetomidine (DEX) exerts its sedative effect by reducing sympathetic tone through α2 adrenergic receptors, and this agent is more selective than its predecessor, the α2 adrenergic receptor agonist clonidine; their α2:α1 ratios are 1620:1 and 220:1, respectively (Virtanen et al., 1988). Unlike most sedatives, DEX has a reversible sedative effect, similar to the unconscious state of natural sleep, and patients can be easily awakened. Therefore, DEX is often used in awake craniotomy or awake sedation. In addition, this drug has anxiolytic and analgesic potential (Lobo et al., 2016; Barends et al., 2017). DEX causes less respiratory depression than traditional sedatives, but there may be an increased risk of hypotension and bradycardia. Notably, the liver is involved in the pharmacokinetics of DEX, so this drug should be administered with caution in patients with liver impairment (Riker et al., 2009; Jakob et al., 2012; Weerink et al., 2017).

**FIGURE 1:**
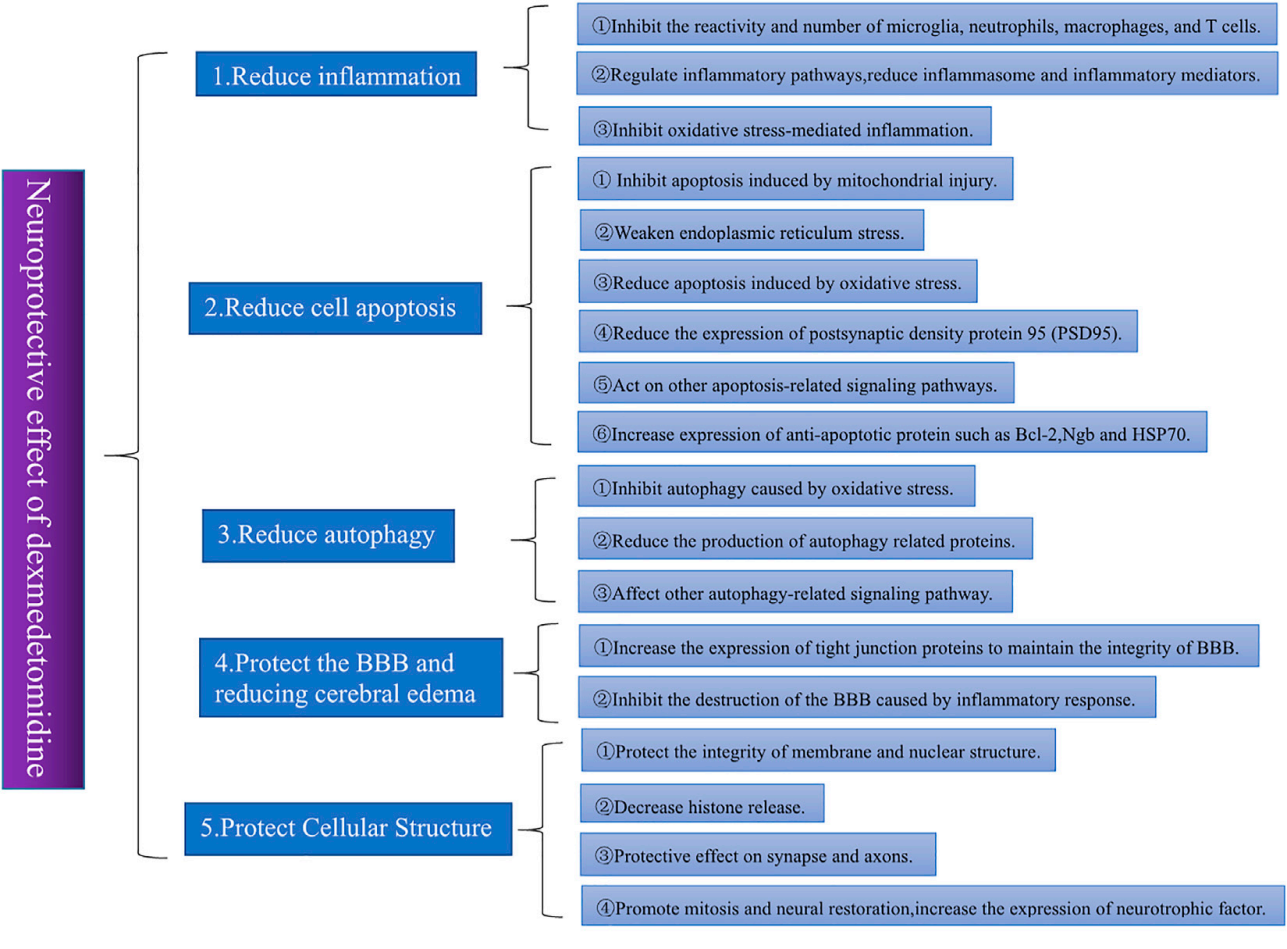
Different trials often offer different explanations for the neuroprotective effects of DEX. By sorting and summarizing relevant studies in recent years, we found that the neuroprotective effects of DEX are mainly reflected in five aspects: 1) reducing inflammation, 2) reducing apoptosis, 3) reducing autophagy, 4) protecting the BBB and reducing cerebral edema, and 5) protecting the cellular structure. Among these aspects, the reduction of the inflammatory response is the most important. Almost all studies on the neuroprotective effect of DEX involve the improvement of nervous system inflammation.

An increasing number of studies have shown that DEX has neuroprotective effects and can reduce the inflammatory response and oxidative stress, inhibit apoptosis, protect the blood–brain barrier (BBB), maintain the balance of the coagulation-anticoagulant system and prevent vasospasm (Wang et al., 2016; Liu et al., 2022). A meta-analysis of 879 patients confirmed the neuroprotective effects of DEX in inhibiting inflammatory responses, reducing neuroendocrine hormone release, and maintaining intracranial homeostasis (Jiang et al., 2017). This literature discusses different neuroprotective mechanisms of DEX. Figure 1 shows the overall framework of the literature.

## Reducing nerve damage caused by inflammation

Recent studies have shown that excessive inflammation in the nervous system is a key factor in the secondary brain damage caused by traumatic brain injury (TBI), subarachnoid hemorrhage (SAH), cerebral ischemia and ischemia–reperfusion (I/R), which mainly manifest as the activation and migration of immune cells, the activation of inflammation-related pathways and the release of inflammatory mediators (Zhang et al., 2013; Miao and Liao, 2014; Van Lieshout et al., 2018).

### DEX can inhibit various types of immune cell infiltration

DEX treatment significantly reduced the number of neutrophils and microglia in damaged nerve tissue (Wang et al., 2018a; Yin et al., 2018; Karakaya et al., 2022). In addition, Wang et al. showed through morphological observation and showed that the transformation of microglia from round to large was slowed, indicating that DEX inhibited microglial reactivity and that microglial reactivity led to the release of inflammatory mediators such as interleukin-1 beta (IL-1β) and activated peripheral migrating macrophages and T cells, leading to inflammatory infiltration of these cells (Mantovani et al., 2014; McKee and Lukens, 2016; Wang et al., 2018a; Yin et al., 2018; Zheng et al., 2018). Activated macrophages can secrete chemokines that promote the accumulation of immune cells such as neutrophils, further exacerbating the inflammatory response (Russo and McGavern, 2016). Tumor necrosis factor receptor superfamily member 5 (CD40) and T-lymphocyte activation antigen CD86 (CD86) are activation markers on the surface of macrophages. Ding et al. showed that DEX treatment could reduce the expression of CD40 and CD86, suggesting its inhibitory effects on macrophage activation and infiltration (Yin et al., 2018; Ding et al., 2019). After several days of TBI, T cells accumulate at the damaged site and are activated by microglia, which may further cause secondary injury. Therefore, T cells play an important role in neuroinflammation (Krämer et al., 2019). Karakaya et al. found that a high concentration of DEX (200 μg/kg) could reduce the amount of T-cell migration occurring approximately 3 days after TBI and reduce T-cell motility (Karakaya et al., 2022).

### DEX can inhibit the generation of inflammatory mediators and inflammasomes

Immune cell activation leads to enhanced activation of inflammation-related pathways and promotes the release of a variety of inflammatory mediators. DEX can act on different processes associated with inflammation-related pathways, ultimately reducing the release of inflammatory mediators and alleviating tissue damage. Toll-like receptor 4 (TLR4) on the surface of microglia plays an important role in regulating the inflammatory response caused by cerebral infarction, cerebral hemorrhage and TBI. Myeloid differentiation primary response 88 (MyD88) is an important component of the TLR4 signaling pathway. When TLR4 is activated, MyD88 activates downstream nuclear factor-kappaB (NF-κB) to produce a series of inflammatory factors that may cause nervous system damage, such as IL-1β, tumor necrosis factor alpha (TNF-α), interleukin-6 (IL-6), and interleukin-18 (IL-18) (Li et al., 2011; Fang et al., 2013; Zhang et al., 2016). Zhu et al. showed that microglia and macrophages play important roles in acute neuroinflammation through the TLR4/MyD88/NF-κB pathway (Zhu et al., 2014). An increase in these inflammatory mediators impairs the BBB, increases neuroinflammation, and induces apoptosis (Zheng and Wong, 2017). IL-1β is the most important inflammatory mediator in the posttraumatic inflammatory response because it peaks within hours of brain tissue injury and promotes the release of other cytokines, prompting nearby microglia to transition from a surveillant to a reactive state and leading to the accumulation of other inflammatory cells (McKee and Lukens, 2016). Many recent studies have shown that DEX can reduce the release of inflammatory mediators, such as IL-1β, TNF-α, IL-6 and monocyte chemotactic protein 1 (MCP-1) by inhibiting TLR4-or NF-κB-related pathways, thus reducing inflammatory damage in the nervous system. Table 1 shows changes in various inflammatory mediators and cells (Wang et al., 2018a; Yin et al., 2018; Li et al., 2019; Feng et al., 2021b; Huang and Hao, 2021).

**TABLE 1:** Changes in various inflammatory mediators and cells.

References	Study model	Subjects	Dose and duration	Results
Wang et al. (2018a)	TBI	C57BL/6J mice	25 μg/kg,3 consecutive days	• The number of neutrophils and microglia↓Microglial reactivity↓IL-1β↓TNF-α↓IL-6↓NF-κB↓NLRP3↓caspase-1↓
Yin et al. (2018)	SAH	Sprague-Dawley (SD) rats	25 μg/kg	• The number of neutrophils, microglia, macrophages and T cells↓IL-1β↓TNF-α↓IL-6↓MCP-1↓TLR4/NF-κB↓NLRP3↓
Zheng et al. (2018)	TBI	SD rats	20 μg/kg, 4 consecutive days	• Microglial reactivity↓ IL-1β↓IL-6↓NLRP3↓caspase-1↓
Ding et al. (2019)	TBI	Humans	0.2–0.3 μg/kg/h, for 100 min	• Macrophage activation↓ IL-1β↓IL-6↓IL-8↓TNF-α↓
		C57BL/6J mice	20 μg/kg	• IL-1β↓IL-6↓IL-8↓CD40↓CD86↓
Karakaya et al. (2022)	TBI	Swiss Albino mice	40 μg/kg or 200 μg/kg	• Microglia and T-cell migration↓NLRP3↓IL-1β↓
Feng et al. (2021b)	TBI	C57BL/6J mice	20 μg/kg	• IL-1β↓IL-6↓TNF-α↓NF-κB↓
Li et al. (2019)	TBI	SD rats	25 μg/kg	• TNF-α↓IL-1β↓IL-6↓NF-κB↓Nrf2↑NQO-1↑HO-1↑
(Huang and Hao, 2021)	TBI	SD rats	Unknown	• TNF-α↓IL-1β↓IL-6↓NF-κB↓NQO-1↑HO-1↑
Sun et al. (2019)	Sepsis	1321N1 astrocytes	1 μM	• NLRP3↓ caspase-1↓
		SD rats	25 μg/kg every 2 h	• caspase-1↓ IL-1β↓IL-18↓
Wang et al. (2022)	Cerebral ischemia	C57BL/6J mice	Loading dose: 1 μg/kg, then 0.05 μg/kg/min for the next 2 h	• iNOS①↓IL-1β↓TNF-α ↓ROS↓ MDA②↓Arg-1↑ IL-4↑ IL-10↑SOD↑ Nrf2↑HO-1↑
		Microglia from C57BL/6J mice	1 μM	• iNOS①↓IL-1β↓TNF-α ↓ROS↓ MDA②↓Arg-1↑ IL-4↑ IL-10↑SOD↑ Nrf2↑HO-1↑
Sun et al. (2021)	Cerebral ischemia	SD rats	Loading dose: 1 μg/kg, then 0.05 μg/kg/min for the next 2 h	• caspase-1↓
		HAPI microglia	1 μM	• caspase-1↓IL-1β↓IL-18↓
Li et al. (2021)	Sunstroke	ICR mice	25 μg/kg	• TNF-α↓IL-1β↓IL-10↑TGF-β↑Neuroprotective microglial phenotype↑
Wang et al. (2018b)	I/R	SD rats	10 μg/kg, 50 μg/kg, or 100 μg/kg	• TNF-α↓IL-1β↓AMPK③↑
Zhu et al. (2019)	I/R	SD rats	Loading dose: 3 μg/kg, then 6 g/kg/h for the next 2 h	• TNF-α↓IL-6↓IL-1β↓
Shen et al. (2017)	TBI	SD rats	15 μg/kg	• TNF-α↓IL-1β↓IL-6↓
Kii et al. (2022)	TBI	C57BL/6 mice and C57BL/6-TG mice	50 μg/kg	• The number of macrophages↓MCP1-CCR2④↓IL-1β↓
Rodríguez-González et al. (2016)	Cerebral ischemia	Astrocytes from SD rats	0.3 μM, 1 μM, or 10 μM	• IL-6↓TNF-α↓
Chen et al. (2017)	Cardiopulmonary bypass (CPB)	SD rats	Loading dose of 2.5 μg/kg or 5 μg/kg before CPB, then a maintenance dose of 2.5 μg/kg/h or 5 μg/kg/h during the CPB	• IL-6↓JAK2/STAT3⑤↓
Tanabe et al. (2014)	IL-1β-induced inflammation	C6 glioma cells	30 µM	• IL-6↓
Qiu et al. (2016)	TBI	SD rats	Loading dose of 3 μg/kg, then 3 μg/kg/min for the next 2 h	• IL-6↓TNF-α↓

①iNOS, is a proinflammatory factor; Arg-1, IL-4, and IL-10, are anti-inflammatory factors (Wang et al., 2022).

②MDA, is an indicator of lipid peroxidation, and SOD, is an important antioxidant enzyme (Xu et al., 2014).

③AMPK, plays an important role in energy metabolism and is thought to alleviate ischemic brain injury(Li et al., 2015).

④MCP1-CCR2 can promote macrophage aggregation, aggravate inflammatory responses and even cause cognitive impairment(Morganti et al., 2015).

⑤Activation of the JAK2/STAT3 pathway alleviates brain injury and inflammatory responses and may be involved in the recovery of neurological function (Oliva et al., 2012; Tao et al., 2015).

The NACHT, LRR and PYD domains-containing protein 3 (NLRP3) inflammasome plays a key role in nervous system inflammation by inducing the expression of immune- and inflammation-related genes. NLRP3 activation requires NF-κB activation, in addition to pathogens or injury-related molecules that initiate the assembly of NLRP3 (Zhou et al., 2016; Yang et al., 2018). Activated NLRP3 further activates caspase-1 and promotes the processing and release of the inflammatory cytokine IL-18, while activated caspase-1 cleaves inactive pro-IL-1β to produce IL-1β, which promotes the inflammatory response (Liu et al., 2013; McKee and Lukens, 2016; Mamik and Power, 2017). Many studies have proven that DEX can reduce the effect of caspase-1 on the precursors of inflammatory mediators by inhibiting the activation of the NLRP3 inflammasome, thus reducing the release of inflammatory mediators and ultimately alleviating neuroinflammation (Wang et al., 2018a; Yin et al., 2018; Zheng et al., 2018; Sun et al., 2019; Karakaya et al., 2022).

### DEX can inhibit oxidative stress-mediated inflammation

Oxidative stress plays an important role in the inflammatory response. Reactive oxygen species (ROS) are one of the most important inflammatory mediators. Nuclear factor erythroid 2-related factor 2 (Nrf2) is an important transcription factor that controls the antioxidant activities of cells, inhibits the increase in intracellular ROS during the inflammatory response and reduces the transformation of macrophages into the proinflammatory M1 phenotype (Wu et al., 2018b; Liu et al., 2018). Nicotinamide adenine dinucleotide phosphate quinone oxidoreductase-1 (NQO-1) and heme oxygenase-1 (HO-1) are important antioxidant enzymes that can alleviate cell damage caused by oxidative stress by promoting the removal of ROS. Li et al. showed that DEX could increase the expression of Nrf2 and its downstream proteins NQO-1 and HO-1 in TBI rat brain tissue (Li et al., 2019). Moreover, HO-1 can also reduce the infiltration of inflammatory cells, while NQO-1 has a neuroprotective effect (Gueron et al., 2009; Sekine et al., 2016; Qu et al., 2020). Additionally, the Nrf2/NLRP3 pathway may play an important role in inhibiting the inflammatory response. Shan et al. examined the mechanism by which DEX alleviates neuropathic pain and accidentally discovered that DEX inhibited the expression of NLRP3 by activating Nrf2, thus reducing the levels of IL-1β and other inflammatory mediators (Shan et al., 2021). *In-vivo* and *in-vitro* experiments by Wang et al. showed that DEX could transform microglial cells from the proinflammatory phenotype to the neuroprotective phenotype by promoting Nrf2 translocation from the cytoplasm to the nucleus and upregulating HO-1 expression. In addition, DEX can reduce the release of inflammatory mediators through the Nrf2/HO-1/NLRP3 pathway (Wang et al., 2022). Mechanistically, heme is broken down by HO-1 into biliverdin and carbon monoxide, which can inhibit NLRP3 inflammasome activation (Gomperts et al., 2017).

## Apoptotic cells are reduced after central nervous system injury

In various nervous system injury models, a large number of apoptotic cells were found at the injury site, and DEX successfully reduced the number of apoptotic nerve cells and affected the expression of apoptosis-related proteins (Kose et al., 2013; Yin et al., 2018; Gao et al., 2019; Huang and Jiang, 2019; Sun et al., 2019; Feng et al., 2021b).

### DEX can affect the expression of anti-apoptotic or pro-apoptotic proteins

The B-cell lymphoma-2 (Bcl-2) protein family plays an important role in the mitochondrial apoptosis pathway, and the proapoptotic protein B-cell lymphoma-2 associated X (Bax) and antiapoptotic protein Bcl-2 are important components of this protein family. The balance between these two proteins maintains the normal process of apoptosis in the physiological state (Youle and Strasser, 2008). Bax promotes apoptosis through three pathways by ①activating other proapoptotic factors; ②inactivating the antiapoptotic protein Bcl-2; and ③triggering the release of corresponding cytokines into the cytoplasm to activate the apoptosis-related protein caspase-3 (Shamas-Din et al., 2011; Zhao et al., 2017). Changes in the levels of caspase-3, which mediates cell apoptosis, can reflect the degree of apoptosis (Wu et al., 2017). A series of studies have shown that DEX can reverse the increase in Bax and the decrease in Bcl-2 after nerve injury and inhibit the level of caspase-3, thus alleviating apoptosis after nerve injury. Zhang et al. showed that DEX can not only affect the expression of these apoptosis-related proteins but also increase the protein expression of heat shock protein 70 (HSP70). HSP70 is a molecular chaperone that can reduce cell damage and has an antiapoptotic effect. Table 2 shows changes in various apoptosis-related proteins (Feder and Hofmann, 1999; Chen et al., 2017; Huang et al., 2018a; Zhang et al., 2018b; Yin et al., 2018; Gao et al., 2019; Li et al., 2019; Feng et al., 2021b; Feng et al., 2021a; Huang and Hao, 2021).

**TABLE 2:** Changes in various apoptosis-related proteins.

References	Study model	Subjects	Dose and duration	Results
Yin et al. (2018)	SAH	SD rats	25 μg/kg	• Bax↓caspase-3↓Bcl-2↑
Feng et al. (2021b)	TBI	C57BL/6J mice	20 μg/kg	• caspase-3↓
Li et al. (2019)	TBI	SD rats	25 μg/kg	• Bax↓Bcl-2↑
(Huang and Hao, 2021)	TBI	SD rats	Unknown	• caspase-3↓Bax↓Bcl-2↑
Sun et al. (2019)	Sepsis	1321N1 astrocytes	1 μM	• ASC①↓GSDMD↓
Chen et al. (2017)	Cardiopulmonary bypass (CPB)	SD rats	Loading dose of 2.5 μg/kg or 5 μg/kg before CPB, then a maintenance dose of 2.5 μg/kg/h or 5 μg/kg/h during CPB	• caspase-3↓
Kose et al. (2013)	Cerebral ischemia	Wistar rats	Loading dose of 3 μg/kg, then 3 μg/kg/h for the next 2 h	• LPO↓
Gao et al. (2019)	I/R	SD rats	50 μg/kg	• Cyt-c↓APAF-1②↓ caspase-3↓Ngb↑HIF-1α/p53↑
(Huang and Jiang, 2019)	Cerebral hemorrhage	C57BL/6 mice	50 μg/kg	• GPX↑SOD↑MDA↓ROS↓PGC-1a↑
Zhang et al. (2018b)	TBI	SD rats	15 μg/kg	• Bax↓Bcl-2↑HSP70↑
Feng et al. (2021a)	Cerebral ischemia	Astrocyte from SD rats	1 μM	• caspase-3↓Bax↓Bcl-2↑JAK/STAT↓JMJD3↓
Huang et al. (2018a)	Cerebral ischemia	SK-N-SH cells	10 μM	• caspase-3↓ survivin③↓ Percentages of G0/G1 -phase and S-phase cells↑ miR-29b↓
Sun et al. (2020)	TBI	C57mice	25 μg/kg or 100 μg/kg	• p-PERK↓p-EIF2α↓ATF4↓CHOP↓IRE1α-ASK1-JNK↓
Li et al. (2018)	TBI	SD rats	20 μg/kg or 50 μg/kg or 100 μg/kg	• PGC-1α↑caspase-3↓MDA↓GPX↑SOD↑
Zhao et al. (2021)	TBI	C57BL/6 mice	100 μg/kg	• PSD95↑PSD95-NR2B-nNOS↓caspase-3↓MMP9↓
Schoeler et al. (2012)	TBI	Hippocampi from C57BL/6 mice	1 μM	• ERK↑

①ASC, is an apoptosis-associated speck-like protein; GSDMD, is a proapoptotic protein (Sun et al., 2019).

②Cyt-c and APAF-1, levels can reflect the degree of damage to hippocampal neuron cells (Gao et al., 2019).

③Survivin is an apoptosis-associated protein(Huang et al., 2018a).

### DEX can reduce apoptosis induced by endoplasmic reticulum stress

When nerve cells are damaged, ischemia, hypoxia or oxidative stress will change the internal environment of the endoplasmic reticulum, causing Ca^2+^ dysregulation in the endoplasmic reticulum, and the increased number of unfolded and misfolded proteins, which triggers endoplasmic reticulum stress, leads to the unfolded protein response (UPR) and ultimately induces apoptosis (Sano and Reed, 2013; Dash et al., 2015). Sun et al. showed that the expression levels of the endoplasmic reticulum stress markers phosphorylated protein kinase RNA-like ER kinase (p-PERK), phosphorylated eukaryotic initiation factor 2α (p-EIF2α), activating transcription factor 4 (ATF4) and C/EBP-homologous protein (CHOP) were significantly increased in TBI mice. CHOP also plays an important role in endoplasmic reticulum pathway-mediated apoptosis, but DEX decreased the levels of these endoplasmic reticulum stress marker proteins. This study suggested that inhibiting the apoptosis-related signaling pathway IRE1α/ASK1/JNK may reduce apoptosis and alleviate nervous system injury (Oyadomari and Mori, 2004; Sun et al., 2020).

### DEX can reduce apoptosis induced by oxidative stress

An increasing number of studies have proven that apoptosis induced by oxidative stress plays an important role in secondary damage in the nervous system (Kose et al., 2013; Li et al., 2018; Huang and Jiang, 2019). Oxidative stress can produce excessive ROS, which can cause damage and impair cell membrane permeability by oxidizing DNA, proteins and membrane lipids and further lead to a decrease in Na^+^-K^+^-ATPase activity on the membrane, which will cause an imbalance in the K^+^ and Mg^2+^ concentrations in the cell. These two ions are important for protein synthesis (White et al., 1993). Kose et al. showed that DEX could reduce lipid peroxidation, and the lipid peroxidation index, protect the cell membrane and membrane receptors, and reduce the damage to the cell structure caused by oxidative stress (Kose et al., 2013). Malondialdehyde (MDA) is an indicator of lipid peroxidation, while superoxide dismutase (SOD) and glutathione peroxidase (GPX) are important antioxidant enzymes. These antioxidant enzymes can convert hydrogen peroxide and lipid peroxides into nontoxic compounds. Huang and Lia et al. suggested that DEX could reduce the apoptosis of nerve cells in patients with brain injury caused by oxidative stress through the proliferator-activated receptor-gamma coactivator 1α (PGC-1α) pathway, and DEX decreased MDA levels while increasing SOD and GPX levels (Xu et al., 2014; Li et al., 2018; Huang and Jiang, 2019).

Zhao et al. (2021) showed that the expression of postsynaptic density protein 95 (PSD95) was decreased in TBI mouse brain tissues, while the levels of the PSD95-N- methyl-D-aspartic acid (PSD95-NMDA) complex were significantly increased. Excessive levels of the PSD95-NMDA complex can lead to the massive release of NO and activate matrix metallopeptidase 9 (MMP9), thus inducing apoptosis, and DEX can inhibit the formation of the PSD95-NMDA complex. In addition, after TBI, a large amount of glutamate is released into the synaptic cleft to activate NMDA receptors and anchor PSD95. This procedure greatly promotes Ca^2+^ influx to produce a large amount of reactive oxygen and active nitrogen, leading to oxidative stress and apoptosis. DEX inhibits the interaction between NMDA and PSD95, thus reducing the oxidative stress response (Gu et al., 2002; Luo et al., 2011).

### DEX can affect the expression of other apoptosis-related signaling pathways and anti-injury factors

Schoeler et al. (2012) examined an *in vitro* TBI model of hippocampal cells and showed that DEX may protect neurons by activating the extracellular regulated kinase (ERK) signaling pathway, which has been shown to protect neuronal cells from mechanical injury-induced apoptosis in previous studies (Ma et al., 2011).

Studies have proven that excessive Jumonji domain-containing protein 3 demethylase (JMJD3) expression can increase the levels of the proapoptotic proteins Bax and caspase-3, leading to cell apoptosis. Feng et al. stimulated astrocytes with oxygen and glucose deprivation (OGD) *in vitro* to simulate ischemic hypoxia after cerebral infarction and demonstrated that DEX could regulate the expression of apoptosis-related proteins by inhibiting the JAK/STAT pathway (which is involved in many crucial biological processes, including cell proliferation, differentiation, apoptosis, and immune regulation) and reducing the expression of JMJD3 (Zhang et al., 2018a; Feng et al., 2021a). Huang et al. used an OGD-induced *in vitro* cerebral ischemia model and showed that DEX could reverse the OGD-induced decreases in the percentages of G0/G1 and S phase cells, increase cell proliferation, reduce cell apoptosis, and inhibit the expression of microRNA-29b (miR-29b) (Huang et al., 2018a). Moreover, the authors demonstrated in another trial that miR-29b could promote neuronal apoptosis by targeting MCL-1 (Huang et al., 2018b).

Neuroglobin (Ngb) is an endogenous anti-injury factor that has a significant protective effect on nerve cells and is thought to play an important role in inhibiting mitochondrial apoptosis: this factor is called the “hemoglobin of the nervous system” (Mammen et al., 2002; Fiocchetti et al., 2021). Gao et al. found that DEX could increase the expression of Ngb and inhibit mitochondrial damage-mediated apoptosis in a rat I/R injury model, possibly by activating the hypoxia-inducible factor 1α (HIF-1α)/p53 signaling pathway. HIF-1α is a regulatory factor that promotes the expression of a series of genes that enable cells to adapt to a hypoxic environment (Demidenko and Blagosklonny, 2011; Gao et al., 2019).

## DEX can affect the autophagy-related signaling pathway to mediate the production of autophagic proteins

Feng et al. (2021b) showed that autophagy was overactivated in the brains of TBI mice, and the autophagy markers Beclin-1 and light chain 3I/II (LC3I/II) were significantly increased, leading to the destruction of the BBB, brain edema, and cell apoptosis. However, DEX significantly reduced Beclin-1 and LC3I/II levels, alleviating autophagy and damage. In mice that were pretreated with the autophagy activator rapamycin, the neuroprotective effect of DEX on reducing autophagy was reversed. In this study, DEX inhibited TBI-induced autophagy by regulating the Nrf2/ROS signaling pathway.

Circlrp1b (a circRNA that regulates autophagy) and DNA-damage regulated autophagy modulator 2 (Dram2) play synergistic roles in autophagy, inflammation and impaired nerve function after TBI, while miR-27a-3p plays the opposite role in these processes. Li et al. showed that the circlrp1b/miR-27a-3p/Dram2 pathway played an important role in autophagy after TBI. DEX decreased the levels of the autophagy-related proteins Dram2, autophagy protein 5 (ATG5), Beclin-1 and LC3I/II, reversing the TBI-induced increased levels of circlrp1b and Dram2 and decreased levels of mir-27a-3p, thus alleviating the autophagic response (Li et al., 2020).

Shen et al. and Zhu et al. showed that DEX could reduce the expression of Beclin-1 and LC3I/II, which are autophagy-related proteins induced by brain injury in rat TBI and I/R models, respectively, and also reduced the degree of edema, vacuolation and autophagosomes, as shown by electron microscopy. The PI3K/Akt pathway plays an important role in cell growth, metabolism and survival, while mechanistic target of rapamycin (mTOR) is the core protein that regulates autophagy. Shen et al. suggested that DEX activated the PI3K/Akt/mTOR signaling pathway to alleviate autophagy (Li et al., 2016; Li et al., 2017; Shen et al., 2017; Zhu et al., 2019). Zhu et al. showed that the reduction in autophagy was due to the inhibition of c-Jun N-terminal kinase (JNK) pathway activation, which was previously thought to be widely involved in stress, cell division, apoptosis and other processes. In the study, Zhu et al. showed that this pathway may also be involved in autophagy (Zhong et al., 2017; Zhu et al., 2019). Table 3 shows changes in various autophagic proteins.

**TABLE 3:** Changes in various autophagic proteins.

References	Study model	Subjects	Dose and duration	Results
Feng et al. (2021b)	TBI	C57BL/6J mice	20 μg/kg	• Beclin-1↓LC3I/II↓Nrf2↑HO-1↑
Zhu et al. (2019)	I/R	SD rats	Loading dose of 3 μg/kg, then 6 g/kg/h for the next 2 h	• Beclin-1↓LC3I/II↓JNK↓
Shen et al. (2017)	TBI	SD rats	15 μg/kg	• p-PI3K/t-PI3K↑p-Akt/t-Akt↑p-mTOR/t-mTOR↑the number of lysosomes↓LC3I/II↓Beclin-1↓
Li et al. (2020)	TBI	SD rats	20 μg/kg, 4 consecutive days	• ATG5↓Beclin-1↓LC3I/II↓circlrp1b/miR-27a-3p/Dram2↓

## DEX can protect the BBB and reducing cerebral edema

BBB injury is a common primary brain injury after TBI. Unavoidable mechanical damage caused by external forces applied to the brain during the acute stage, which can lead to brain edema, further increasing intracranial pressure and worsening patient prognosis (Zhao et al., 2016).

### DEX can increase the expression of tight junction proteins

Tight junction proteins, including occludin, zona occludens-1 (ZO-1) and claudin-5, are important components of the BBB, and damage to the BBB is accompanied by the degradation of tight junction proteins. Wang et al. and Shen et al. showed that DEX treatment could increase the expression of tight junction proteins to maintain the integrity of the BBB (Shen et al., 2017; Wang et al., 2018a). As the expression of tight junction proteins increased, the permeability of the BBB was restored. Experimentally, BBB permeability and damage are often assessed by observing the extravasation of Evans blue dye. A series of studies have shown that DEX can significantly reduce the leakage of Evans blue in the cerebral cortex. Reversing the increase in BBB permeability caused by TBI reduces brain edema (Shen et al., 2017; Wang et al., 2018a; Sun et al., 2020; Feng et al., 2021b). In addition, Yin et al. used a model of SAH and showed that DEX alleviated brain edema by increasing tight junction protein expression and reducing the extravasation of Evans blue dye to indicate a protective effect on the BBB (Yin et al., 2018). Table 4 shows changes in various tight junction proteins.

**TABLE 4:** Changes in brain water content and various tight junction proteins.

References	Study model	Subjects	Dose and duration	Results
Wang et al. (2018a)	TBI	C57BL/6J mice	25 μg/kg,3 consecutive days	• Brain water content↓ Evans blue dye extravasation↓ZO-1↑occludin↑
Yin et al. (2018)	SAH	Sprague-Dawley (SD) rats	25 μg/kg	• Brain water content↓ Evans blue dye extravasation↓ZO-1↑occludin↑
Feng et al. (2021b)	TBI	C57BL/6J mice	20 μg/kg	• Brain water content↓ Evans blue dye extravasation↓
Shen et al. (2017)	TBI	SD rats	15 μg/kg	• Brain water content↓ZO-1↑claudin-5↑
Sun et al. (2020)	TBI	C57mice	25 μg/kg or 100 μg/kg	• Brain water content↓Evans blue dye extravasation↓

### DEX can alleviate inflammation-mediated BBB injury

In addition to acute mechanical injury, the inflammatory response after TBI also plays an important role in BBB disruption. A growing body of research suggests that an excessive inflammatory response after brain injury can lead to BBB breakdown and nerve damage (Abdul-Muneer et al., 2015; Gao et al., 2015). Peripheral neutrophils, macrophages and other inflammatory cells can migrate to the brain through the damaged BBB, further exacerbating the inflammatory response (Xu et al., 2017). DEX can reduce the expression of the inflammatory mediators IL-1β, TNF-α, IL-6 and NLRP3 inflammasome in the brain after TBI (Wang et al., 2018a; Feng et al., 2021b; Li et al., 2021). Shen et al. suggested that DEX could not only reduce the inflammatory response but also alleviate BBB injury after TBI by activating the PI3K/Akt/mTOR signaling pathway, which may increase the expression of the tight junction proteins occludin, claudin-1 and ZO-1 (Shen et al., 2017).

## Cellular structure protection and enhanced expression of genes related to neuroprotection

### DEX can protect the integrity of the astrocyte structure

In addition, DEX has been shown to play an important role in protecting cellular structures after nerve injury. Sun et al. found that DEX could protect the integrity of the astrocyte membrane and nuclear structure, and this type of cell provides support for neurons through glial transmission, synaptic remodeling, gap connections, energy metabolism, information transmission and other processes (Rama Rao and Kielian, 2015; Sun et al., 2019). In this study, abnormally elevated nuclear histones were detected in the cytoplasm during cell injury, and the cytoskeletal structure was destroyed. However, DEX significantly improved these outcomes, and the number of histones released from the nucleus was significantly reduced. The overall cytoskeletal structure of the cells was relatively intact, maintaining normal cellular morphology. In addition, these findings showed that histones released from the nucleus can cause neuronal damage (Sun et al., 2019).

### DEX can reduce synaptic and axonal damage

Synaptic damage often leads to neuronal dysfunction and even apoptosis after TBI. PSD95 plays a supporting role in synaptic development, promotes improvements in synaptic function, and plays a role in synaptic integration and functional recovery when nerve cells are damaged (Keith and El-Husseini, 2008; Merlo et al., 2014; Mo et al., 2016). Zhao et al. showed that the expression of PSD95 was significantly decreased after TBI, while DEX significantly reversed this outcome. Mechanistically, DEX reduces the production of PSD95-NMDA compounds, which has a negative effect on the repair of damaged synapses and recovery from cognitive impairment (Zhao et al., 2021). Wu et al. used an anti-synaptophysin antibody to immunostain samples and found that the intensity and number of synaptophysin-positive cells after TBI were lower than those in the control group, indicating synaptic degeneration, and DEX treatment protected synapses by increasing the intensity of synaptophysin staining (Wu et al., 2018a). In addition, this study showed a protective effect of DEX on axons. β-amyloid precursor protein (β-APP) is an important marker of axonal injury because β-APP can be rapidly transported in normal axons. When axons are damaged, β-APP transport is impaired, resulting in the accumulation of β-APP (Kaur et al., 1999). Therefore, axonal injury can be assessed by β-APP immunostaining. Compared with the control treatment, DEX treatment significantly weakened β-APP immunoreactivity, suggesting that DEX could alleviate the axonal injury caused by TBI (Wu et al., 2018a). Table 5 shows the protective effect of DEX on the cell structure.

**TABLE 5:** Protective effect of DEX on the cell structure.

References	Study model	Subjects	Dose and duration	Results
Sun et al. (2019)	Sepsis	1321N1 astrocytes	1 μM	• Nuclear histones↓ Stability of cytoskeletal structure↑
Li et al. (2021)	Sunstroke	ICR mice	25 μg/kg	• TREM2①↑Arg-1↑
Rodríguez-González et al. (2016)	Cerebral ischemia	Astrocytes from SD rats	0.3 μM or 1μ M or 10 μM	• BDNF↑
Chen et al. (2017)	Cardiopulmonary bypass (CPB)	SD rats	Loading dose of 2.5 μg/kg or 5 μg/kg before CPB, then a maintenance dose of 2.5 μg/kg/h or 5 μg/kg/h during the CPB	• S100β↓NSE②↓
Zhao et al. (2021)	TBI	C57BL/6 mice	100 μg/kg	• PSD95↑PSD95-NMDA↓
Wu et al. (2018a)	TBI	C57BL/6 mice	1 μg/kg or 10 μg/kg or 100 μg/kg	• Intensity and number of synaptophysin-positive cells↑β-APP↓
Yang et al. (2021)	TBI	SD rats	100 μg/kg	• Lyn↑Cdk1↑miR-7a-5p↓miR-873-5p③↓
Shi et al. (2018)	I/R	SD rats	Loading dose of 3 μg/kg, then 6 μg/kg/h for the next 2 h	• ERK1/2↑CREB↑ADRA2A④↑
	I/R	Astrocytes from SD rats	500 ng/ml for 3 h	• ERK1/2↑CREB↑ADRA2A④↑
Teng et al. (2019)	I/R	SD rats	1 μg/kg/d,7 consecutive days	• ERK1/2↑CREB↑

①TREM2 is a glycoprotein receptor for microglia that is involved in the regulation of neuroinflammation(Neumann and Takahashi, 2007).

②Changes in S100β and NSE, levels can reflect the degree of brain injury and are specific markers of central nervous system injury (Luo et al., 2016).

③Downregulation of miR-7a-5p and miR-873-5p is related to the neuroprotective effect of DEX(Yang et al., 2021).

④ADRA2A-mediated ERK1/2 phosphorylation plays a neuroprotective role(Shi et al., 2018).

## Other neuroprotective effects of DEX

Extracellular regulated kinase1/2 (ERK1/2) is an indispensable mediator of mitosis, and cyclic adenosine monophosphate response element binding protein (CREB) maintains the survival of damaged neurons and promotes the subsequent regeneration and repair process. Teng et al. and Shi et al. showed that DEX could increase the expression of ERK1/2 and CREB in damaged nerve cells (Cao et al., 2012; Shi et al., 2018; Teng et al., 2019). Yang et al. compared mRNA and miRNA expression in TBI model rats in the control group and DEX treatment group by RNA sequencing and conducted bioinformatics analysis: the results showed that Lyn and Cdk1 may be central genes involved in DEX-mediated neuroprotection (Yang et al., 2021). Cdk1 is critical in mitotic substrate processing, and Lyn regulates B-cell signaling pathways to maintain tolerance to autoantigens. In addition, activation of the Lyn-ERK1/2-CREB pathway increases the expression of brain-derived neurotrophic factor (BDNF) (Zhang et al., 2010; Malumbres, 2014; Brodie et al., 2018). Rodríguez et al. and Degos et al. showed that DEX could increase the expression of BDNF in astrocytes to play a neuroprotective role (Degos et al., 2013; Rodríguez-González et al., 2016).

## Discussion

**FIGURE 2:**
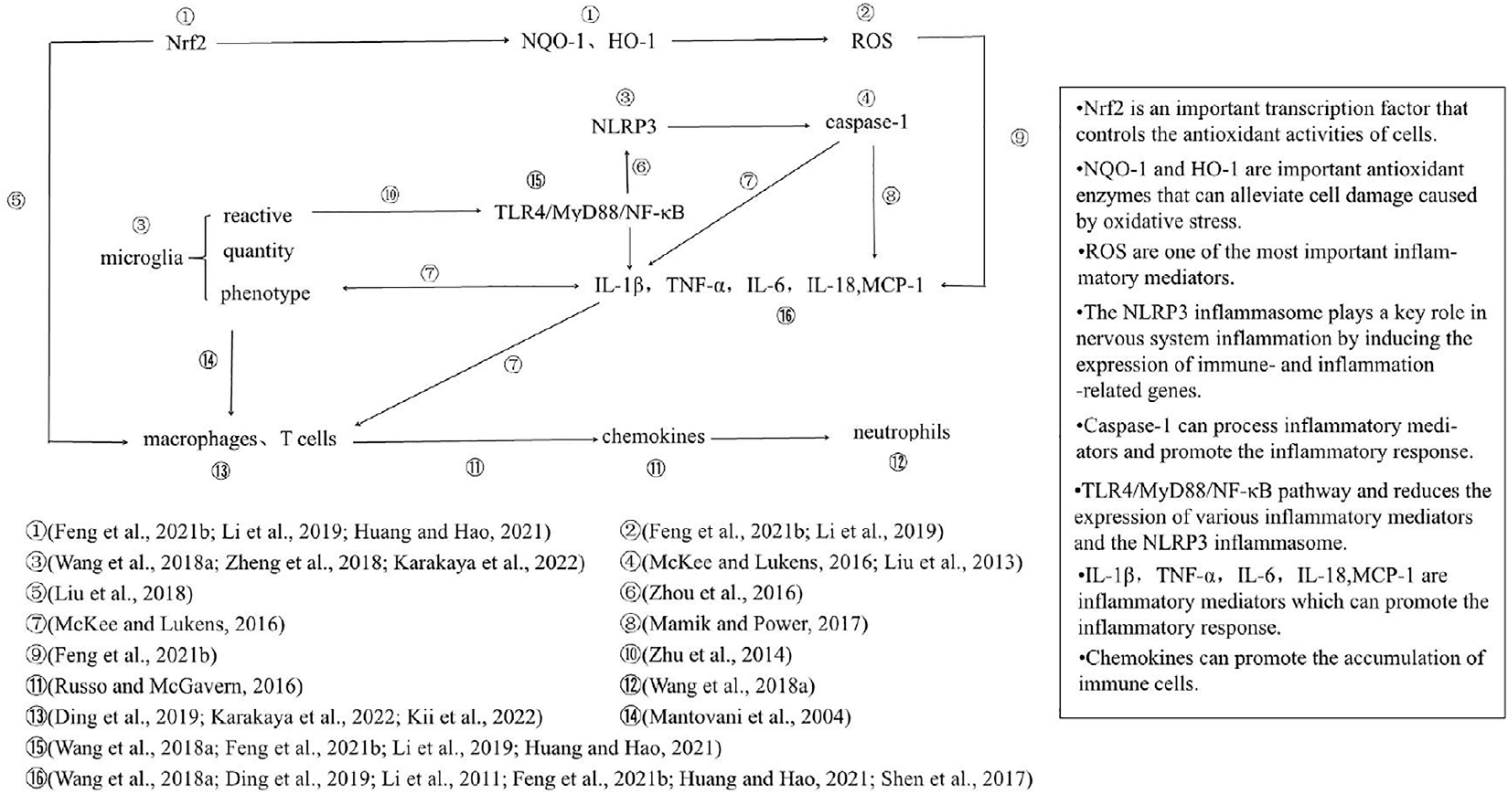
The TBI model plays a leading role in research on the neuroprotective effect of DEX. Therefore, trials with TBI as the model were screened and summarized to explore the neuroprotective mechanism of DEX under the same injury conditions. In the TBI model, the action of DEX involves two main processes, the inflammatory response and oxidative stress, which act on multiple levels of cells, signaling pathways and molecules; oxidative stress process ultimately promotes the release of inflammatory mediators and infiltration of inflammatory cells. Once again, it was confirmed that DEX plays an important role in alleviating inflammation in patients with neurological injury.

The effects of DEX on inflammation after TBI are summarized in Figure 2. At the cellular level, DEX reduces the density of microglial cells, inhibits microglial reactivity, and reduces inflammatory cell infiltration by inhibiting the reactivity of microglia and the accumulation of macrophages and T cells (Mantovani et al., 2004; Wang et al., 2018a; Zheng et al., 2018; Ding et al., 2019; Karakaya et al., 2022; Kii et al., 2022). At the molecular level, DEX inhibits the microglial TLR4/MyD88/NF-κB pathway and reduces the expression of various inflammatory mediators and the NLRP3 inflammasome (Wang et al., 2018a; Zheng et al., 2018; Li et al., 2019; Feng et al., 2021b; Huang and Hao, 2021; Karakaya et al., 2022). The oxidative stress process mediates inflammation through ROS and promotes the infiltration of macrophages (Liu et al., 2018; Kii et al., 2022). Therefore, we believe that the effect of DEX on microglia plays an important role in alleviating inflammatory injury in TBI, possibly by inhibiting the TLR4/MyD88/NF-κB/NLRP3 pathway. Based on these studies, we found that DEX could affect most of the inflammatory processes shown in Figure 2, but DEX-mediated inhibition of microglia and the promotion of Nrf2 expression may be the initial links in its neuroprotective effect (Wang et al., 2018a; Zheng et al., 2018; Li et al., 2019; Feng et al., 2021b; Huang and Hao, 2021; Karakaya et al., 2022). Recent studies have shown that these two processes can drive all other processes after being initiated by DEX. However, the direct effect of DEX on other processes cannot be ruled out. Notably, in models of ischemic brain injury and neuralgia, DEX acts on oxidative stress processes to reduce the number of inflammatory microglia and NLRP3 inflammasome formation. This result suggests that the oxidative stress process may be directly linked to the inflammatory response through microglia and the NLRP3 inflammasome (Shan et al., 2021; Wang et al., 2022). However, it is undeniable that there may be some heterogeneity in the mechanism of inflammation due to different experimental models.

DEX decreases apoptosis mainly by inhibiting the mitochondrial pathway of apoptosis; mitigating endoplasmic reticulum stress and oxidative stress; and decreasing the expression of proapoptotic proteins. According to the literature, these three apoptosis pathways are relatively independent, and there is no evidence to verify a connection between them. The mechanism by which DEX inhibits mitochondrial apoptosis may involve reducing the expression of JMJD3 by inhibiting the JAK/STAT pathway, reducing the production of the proapoptotic proteins Bax and caspase-3, and activating the HIF-1α/p53 signaling pathway to enhance the adaptation of nerve cells to adverse environments (Gao et al., 2019; Feng et al., 2021a). The DEX-mediated inhibition of endoplasmic reticulum stress pathway-mediated apoptosis may be related to inhibiting the IRE1α-ASK1-JNK pathway (Sun et al., 2020). DEX may inhibit oxidative stress through the PGC-1α pathway and the interaction between NMDA and PSD95 (Li et al., 2018; Zhao et al., 2021).

**FIGURE 3:**
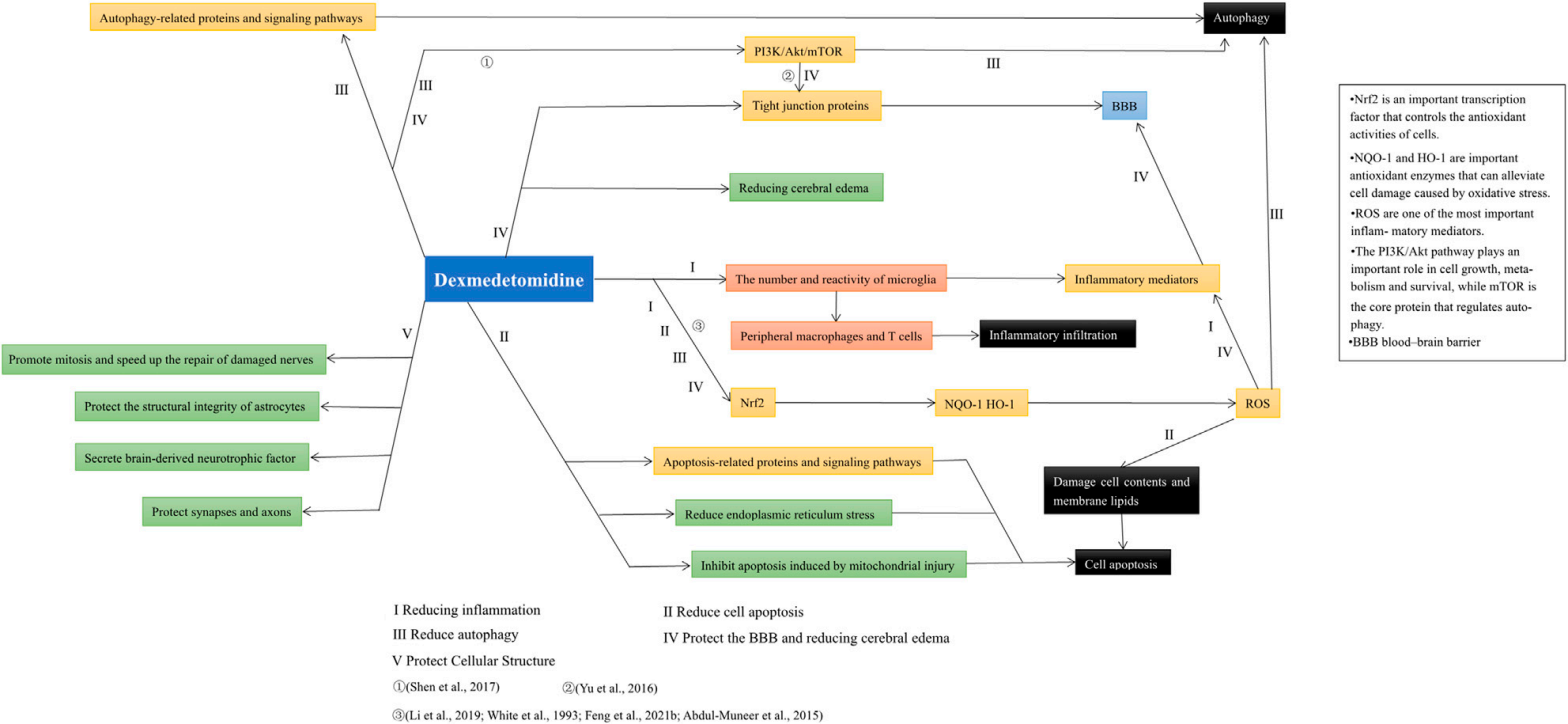
After summarizing the literature included in this paper, it was found that the neuroprotective effects of dexmedetomidine are not completely independent. Its effect on Nrf2 signaling pathway can reduce inflammation, apoptosis, autophagy, and protect BBB by inhibiting ROS generation. Additionally, the PI3K/Akt/mTOR pathway plays an important role in alleviating autophagy and protecting the BBB.

Based on recent studies, we expounded on the role and possible mechanism by which DEX alleviates autophagy, protects the BBB and maintains normal cellular structure. In addition, we found that the various protective effects of DEX on the nervous system were not independent. For example, inhibiting oxidative stress can reduce autophagy and apoptosis, while reducing inflammation can prevent the destruction of the BBB (Wang et al., 2018a; Feng et al., 2021b). DEX activates the PI3K/Akt/mTOR signaling pathway and plays an active role in alleviating autophagy and protecting the BBB (Shen et al., 2017). The neuroprotective effect of dexmedetomidine and its relations in various aspects are shown in Figure 3. In addition, the protective effect of DEX in patients with brain injury is not limited to neuroprotection. For example, studies have shown that DEX can reduce delirium and agitation in patients after TBI (Roberson et al., 2021; Soltani et al., 2021), and DEX can also reduce paroxysmal sympathetic hyperactivity (PSH) caused by nervous system damage (Tang et al., 2017; Branstetter et al., 2020).

This review indicates that DEX can play a neuroprotective role in different aspects of brain injury caused by a variety of factors and that DEX can significantly improve patient prognosis. However, this study has some limitations. First, most of the current experiments on the neuroprotective effects of DEX are animal experiments or *in vitro* cell experiments, and clinical data are lacking. In addition, priority is given to TBI and cerebral ischemia models; few other models of neurological damage are available for review. Therefore, in this paper, the goal was not to differentiate damage types but to examine neuroprotective effects; different types of nerve damage have certain similarities. Further understanding of the neuroprotective effect of DEX on different injury types will be important in the future.

## Glossary

Akt: V-akt murine thymoma viral oncogene homolog
AMPK: AMP-activated protein kinase
APAF-1: apoptotic protease activating factor 1
Arg-1: arginase-1
ASC: apoptosis-associated speck-like protein containing a CARD
ASK1: apoptosis signal-regulating kinase 1
ATF4: activating transcription factor 4
ATG5: autophagy protein 5
Bax: B-cell lymphoma-2 associated X
BBB: blood–brain barrier
Bcl-2: B-cell lymphoma-2
BDNF: brain-derived neurotrophic factor
CCR2*: C-C chemokine receptor type 2
CD40*: tumor necrosis factor receptor superfamily member 5
CD86*: T-lymphocyte activation antigen CD86
Cdk1: cyclin-dependent kinase 1
CHOP: C/EBP-homologous protein
CREB: cyclic adenosine monophosphate response element binding protein
Cyt-c: cytochrome c
DEX: dexmedetomidine
Dram2: DNA-damage regulated autophagy modulator 2
ERK: extracellular regulated kinase
GPX: glutathione peroxidase
GSDMD: gasdermin D
HIF-1α: hypoxia-inducible factor-1α
HO-1: heme oxygenase-1
HSP70: heat shock protein 70
I/R: ischemia–reperfusion
IL-1β: interleukin-1beta
IL-4: interleukin-4
IL-6: interleukin-6
IL-8: interleukin-8
IL-10: interleukin-10
IL-18: interleukin-18
iNOS: inducible nitric oxide synthase
IRE1α: inositol-requiring enzyme 1α
JAK2: Janus kinase 2
JMJD3: Jumonji domain-containing protein 3 demethylase
JNK: c-Jun N-terminal kinase
LC3I/II: light chain 3I/II
LPO: lipid peroxidation
MCL-1: myeloid cell leukemia-1
MCP-1: monocyte chemotactic protein 1
MDA: malondialdehyde
miR-29b: microRNA-29b
MMP9: matrix metallopeptidase 9
mTOR: mechanistic target of rapamycin
MyD88: myeloid differentiation primary response 88
NF-κB: nuclear factor-kappaB
Ngb: neuroglobin
NLRP3*: NACHT, LRR and PYD domains-containing protein 3
NMDA: N-methyl-D-aspartate
nNOS: neuronal nitric oxide synthase
NQO-1: nicotinamide adenine dinucleotide phosphate quinone oxidoreductase-1
NR2B: N-methyl-D-aspartic acid receptor 2B
Nrf2*: nuclear factor erythroid 2-related factor 2
OGD: oxygen and glucose deprivation
p-EIF2α: phosphorylated eukaryotic initiation factor 2α
PGC-1α: proliferator-activated receptor-gamma coactivator 1α
PI3K: phosphoinositide 3-kinase
p-PERK: phosphorylated protein kinase RNA-like ER kinase
PSD95: postsynaptic density protein 95
PSH: paroxysmal sympathetic hyperactivity
ROS: reactive oxygen species
SAH: subarachnoid hemorrhage
SOD: superoxide dismutase
STAT3: signal transducer and activator of transcription 3
TBI: traumatic brain injury
TGF-β: transforming growth factor-β
TLR4: Toll-like receptor 4
TNF-α: tumor necrosis factor alpha
UPR: unfolded protein response
ZO-1: zona occludens-1
β-APP: β-amyloid precursor protein
